# Modeling the Impact of Viscosity on Fricke Gel Dosimeter Radiolysis: A Radiation Chemical Simulation Approach

**DOI:** 10.3390/gels11070489

**Published:** 2025-06-24

**Authors:** Sumaiya Akhter Ria, Jintana Meesungnoen, Jean-Paul Jay-Gerin

**Affiliations:** Department of Medical Imaging and Radiation Sciences, Faculty of Medicine and Health Sciences, Université de Sherbrooke, 3001, 12th Avenue Nord, Sherbrooke, QC J1H 5N4, Canada; sumaiya.akhter.ria@usherbrooke.ca (S.A.R.); jintana.meesungnoen@usherbrooke.ca (J.M.)

**Keywords:** Fricke gel dosimeter, radiolysis, Fricke yield, viscosity, diffusion, linear energy transfer (LET), Monte Carlo track chemistry simulations

## Abstract

The Fricke gel dosimeter, a hydrogel-based chemical dosimeter containing dissolved ferrous sulfate, measures 3D radiation dose distributions by oxidizing Fe^2+^ to Fe^3+^ upon irradiation. This study investigates the variation in Fricke yield, *G*(Fe^3+^), from a radiation–chemical perspective in both standard and gel-like Fricke systems of varying viscosities, under low- and high-linear energy transfer (LET) conditions. We employed our Monte Carlo track chemistry code IONLYS-IRT, using protons of 300 MeV (LET~0.3 keV/µm) and 1 MeV (LET~25 keV/µm) as radiation sources. To assess the impact of viscosity on *G*(Fe^3+^), we systematically varied the diffusion coefficients of all radiolytic species in the Fricke gel, including Fe^2+^ and Fe^3+^ ions. Increasing gel viscosity reduces Fe^3+^ diffusion and stabilizes spatial dose distributions but also lowers *G*(Fe^3+^), compromising measurement accuracy and sensitivity—especially under high-LET irradiation. Our results show that an optimal Fricke gel dosimeter must balance these competing factors. Simulations with lower sulfuric acid concentrations (e.g., 0.05 M vs. 0.4 M) further revealed that *G*(Fe^3+^) values at ~100 s are nearly identical for both low- and high-LET conditions. This study underscores the utility of Monte Carlo simulations in modeling viscosity effects on Fricke gel radiolysis, guiding dosimeter optimization to maximize sensitivity and accuracy while preserving spatial dose distribution integrity.

## 1. Introduction

The ferrous sulfate dosimeter, commonly known as the Fricke dosimeter after Hugo Fricke [1,2,3], consists of an air-saturated (~2.5 × 10^−4^ M O_2_) aqueous solution composed of 1 mM ferrous sulfate in 0.4 M sulfuric acid (pH~0.46) [4,5]. Renowned for its accuracy, reproducibility, and linear dose response [6,7,8], it has long been a cornerstone of radiation chemistry and dosimetry [4,9]. This system reliably measures radiation dose by tracking the oxidation of ferrous (Fe^2+^) to ferric (Fe^3+^) ions (e.g., [4,5,9,10,11] and references therein).

The efficiency of this oxidation, quantified by the Fricke yield (*G*-value), is influenced by various factors [4,5]. A primary focus of this study is to investigate how increased viscosity—and the resulting reduction in molecular diffusion—affects the radiolytic behavior of the Fricke dosimeter, especially when the solution is transformed into a more viscous or gel-like medium. The diffusion of Fe^2+^ and Fe^3+^ ions, along with radiolytic species such as ^•^OH radicals, H^•^ atoms, and hydrogen peroxide, plays a critical role in determining the efficiency of radiation-induced oxidation and, consequently, the dosimetric response. This effect is particularly relevant in Fricke gel dosimeters (e.g., [12,13,14,15,16,17,18,19] and references therein), which incorporate the aqueous Fricke solution into a gel matrix. In such systems, diffusion coefficients are influenced by factors such as the gel type (e.g., gelatin or agarose), concentration, temperature, and pH.

Another key factor examined in this study is the linear energy transfer (LET) of radiation. High-LET radiation tends to reduce the dosimeter’s sensitivity by yielding lower Fricke *G*-values [5,9,20]. Additionally, the occurrence of a “Bragg peak”—the region where the density of ionization increases as the ionizing particle approaches the end of its track (range) [21,22]—as observed in proton and heavy-ion therapies, poses further challenges when using Fricke gel dosimeters. These gels are well-suited for capturing the steep dose gradients at the Bragg peak and its rapid dose fall-off. However, to preserve the sharpness of this transition, the gel matrix must be carefully optimized. In particular, viscosity must be sufficient to limit ferric ion diffusion, which can smear the dose distribution post-irradiation and compromise the accuracy of 3D dose mapping near the Bragg peak.

Despite the aforementioned limitations, Fricke gel dosimeters offer high spatial resolution and excellent water and tissue equivalence, making them well-suited for complex radiation fields [17,18,19]. These properties support their use in high-precision radiotherapy, where accurate 3D dose mapping is essential for effective tumor targeting and minimizing exposure to surrounding healthy tissue.

In this study, we present Monte Carlo simulations exploring the impact of viscosity on the radiolysis of Fricke gel dosimeters under both low- and high-LET proton irradiation, using 300 MeV (LET~0.3 keV/µm) and 1 MeV (LET~25 keV/µm) protons as illustrative examples. From a radiation–chemical perspective, we model variations in Fe^3+^ ion yield by systematically adjusting the diffusion coefficients of all relevant radiolytic species in the gel, including Fe^2+^ and Fe^3+^ ions, to reflect different viscosities. The novelty of our approach lies in integrating Monte Carlo track chemistry with key practical challenges in gel dosimetry. The results provide quantitative insights directly applicable to the design and optimization of Fricke gel dosimeters. Furthermore, given that concentrated sulfuric acid (0.4 M) can degrade the gel matrix, we also examine the use of a reduced H_2_SO_4_ concentration (0.05 M), commonly employed in gel formulations to preserve structural integrity.

## 2. Results and Discussion

### 2.1. Radiolysis of Deaerated 0.4 M H_2_SO_4_ Aqueous Solutions: Formation of Primary Radical and Molecular Products

Most radiolysis experiments on acidic aqueous solutions were carried out as early as the 1950s, preceding those conducted in neutral media (e.g., [5,23]). Sulfuric acid was commonly used at a concentration of 0.4 M (pH~0.46) [9,24]. Notably, the pioneering work of Fricke [1,2,3,4] in developing the ferrous sulfate dosimeter underpinned many of these early investigations.

For low-LET radiations—such as Compton electrons from ^60^Co γ-rays, fast electrons, or several hundred MeV protons (LET~0.3 keV/µm)—the initial radiation track consists of small, well-separated, nearly spherical “spurs”, which are localized clusters of radiolytic species [25,26]. In the absence of dose–rate effects, these spurs evolve by diffusion, as described by Fick’s laws, and typically coalesce within ~0.2 µs [27]. Once merged, the radiation track effectively dissipates, and the radiolytic species that escape spur reactions become homogeneously distributed throughout the bulk solution.

The main reactive species present after spur coalescence are e^−^_aq_, H^•^, ^•^OH, H_2_, and H_2_O_2_—commonly referred to as “radical” and “molecular” products [9,23,24,28,29,30,31]. These “primary” species have corresponding “escape” yields—*g*(e^−^_aq_), *g*(H^•^), *g*(^•^OH), *g*(H_2_), and *g*(H_2_O_2_)—which quantify the number of species formed or consumed per 100 eV of absorbed radiation. These values represent the amounts available to react with solutes (e.g., Fe^2+^ ions in the Fricke dosimeter, the focus of this study) in dilute aqueous solutions [9]. *G* values are reported in molecules per 100 eV; to convert to SI units: 1 molecule/100 eV ≈ 1.0364 × 10^−7^ mol/J. Free radicals are indicated by a “dot” (^•^) at the position of the unpaired electron in their chemical formula.

The accepted primary yields of radical and molecular products from air-free ^60^Co γ-irradiation of 0.4 M H_2_SO_4_ aqueous solutions at 25 °C are [32]:

*g*(e^−^_aq_) = 0    *g*(H^•^) = 3.70    *g*(H_2_) = 0.40 *g*(^•^OH) = 2.90 *g*(H_2_O_2_) = 0.80 *g*(HO_2_^•^) = 0.02 (1)

Here, HO_2_^•^ (hydroperoxyl radical) is the conjugate acid of O_2_^•−^ (superoxide anion radical; p*K*_a_ = 4.8 at 25 °C) [33] and constitutes a minor radiolytic product, formed only in trace amounts [34]. A review of available data indicates that these yields remain consistent across solutions of similar pH, regardless of anion type [24].

### 2.2. The Radiation Chemistry of the Standard (Air-Saturated) Fricke Dosimeter

The Fricke dosimeter is the most widely used and well-characterized liquid chemical dosimeter. It is easy to prepare and allows straightforward quantification of energy deposited by ionizing radiation. Its chemistry is based on the oxidation of ferrous to ferric ions by ^•^OH, HO_2_^•^, and H_2_O_2_ formed during radiolysis of acidic, air-saturated water. The reaction scheme is as follows [4,5,9,10,11,20]:

(2) $$\begin{array}{cc} {e^{-}}_{aq}+H_{3}O^{+}\;\to \;H^{•}+H_{2}O & k=2.1\times 10^{10}\;M^{-1}\;s^{-1}\\ & {pK}_{a}\;(H^{•}/{e^{-}}_{aq})=9.74 \end{array}\;$$

H^•^ + O_2_ → HO_2_^•^    *k* = 1.3 × 10^10^ M^−1^ s^−1^ (3)

^•^OH + Fe^2+^ → Fe^3+^ + OH^−^  *k* = 3.4 × 10^8^ M^−1^ s^−1^ (4)

HO_2_^•^ + Fe^2+^ → Fe^3+^ + HO_2_^−^  *k* = 7.9 × 10^5^ M^−1^ s^−1^ (5)

(6) $$\begin{array}{cc} {HO}_{2^{-}}+H^{+}\;\to \;H_{2}O_{2} & k=5.0\times 10^{10}\;M^{-1}\;s^{-1}\\ & {pK}_{a}\;(H_{2}O_{2}/{HO}_{2^{-}})=11.84 \end{array}\;$$

H_2_O_2_ + Fe^2+^ → Fe^3+^ + ^•^OH + OH^−^ *k* = 52 M^−1^ s^−1^ (7)

The rate constants (*k*) given for reactions between ions are at infinite dilution (zero ionic strength).

The ferric ion yield, *G*(Fe^3+^), is linked to the “escape” radical and molecular yields in Equation (1) and is expressed by the following stoichiometric equation [9,10]:

*G*(Fe^3+^)_aerated_ = *g*(^•^OH) + 3 *g*(H^•^) + 2 *g*(H_2_O_2_) + 3 *g*(HO_2_^•^) (8)

Using the yields from Equation (1), the calculated *G*(Fe^3+^)_aerated_ agrees within 1–2% of the observed value of 15.5 ± 0.2 ions/100 eV for ^60^Co γ-rays or fast electrons (LET~0.3 keV/µm) [4,6,7,8,9].

In 0.4 M H_2_SO_4_ solutions, a small fraction of ^•^OH radicals react with HSO_4_^−^ to form the sulfate radical SO_4_^•−^ (or its protonated form HSO_4_^•^; p*K*_a_ = 1.9) [20]:

^•^OH + HSO_4_^−^ → H_2_O + SO_4_^•−^  *k* = 1.5 × 10^5^ M^−1^ s^−1^ (9)

However, this does not affect the overall ferric ion yield of Equation (8), as SO_4_^•−^ is stoichiometrically equivalent to ^•^OH [35]:

Fe^2+^ + SO_4_^•−^ → Fe^3+^ + SO_4_^2−^  *k* = 9.9 × 10^8^ M^−1^ s^−1^ (10)

### 2.3. Time Evolution of G(Fe^3+^) in the Radiolysis of the Fricke Dosimeter—LET Effects

**Figure 1:**
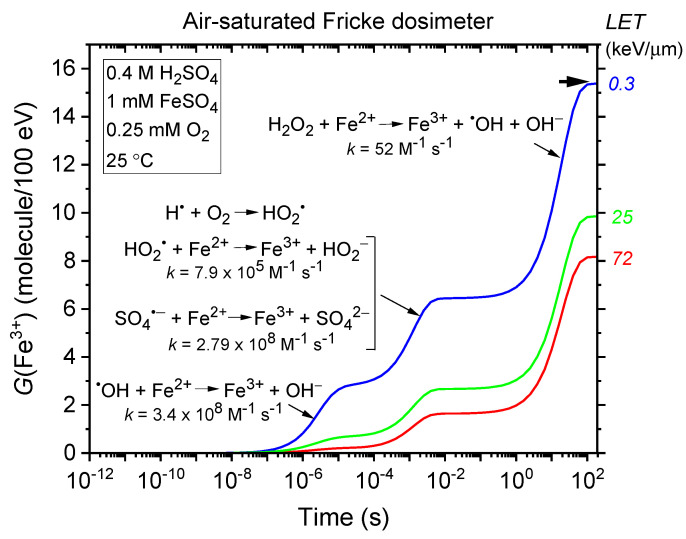
Time evolution of *G*(Fe^3+^) from Monte Carlo track chemistry simulations of the radiolysis of an air-saturated Fricke solution (1 mM FeSO_4_ in 0.4 M H_2_SO_4_), irradiated with 300 MeV protons (LET~0.3 keV/µm) (blue line), 1 MeV protons (LET~25 keV/µm) (green line), and 150 keV protons (LET~72 keV/µm) (red line), over a time range of ~1 ps to 200 s (see Section 4). The dissolved oxygen concentration was set at 2.5 × 10^−4^ M. The arrow on the top right indicates the recommended *G*(Fe^3+^) value for the air-saturated Fricke dosimeter irradiated with ^60^Co γ-rays or fast electrons (15.5 ± 0.2 molecules/100 eV). Under the same low-LET conditions, our computed *G*(Fe^3+^) value at ~100 s is ~15.35 molecules/100 eV, which aligns closely with the recommended value. Notably, the error bars are minimal, well below a few percent, and fall within the width of the drawing lines.

The kinetics of Fe^3+^ formation in the standard (air-saturated) Fricke dosimeter are well-documented (e.g., [10,20,36,37] and references therein). *G*(Fe^3+^) is time-dependent, reflecting the differing reaction times between Fe^2+^ and the reactive species ^•^OH (and, to a lesser extent, SO_4_^•−^), HO_2_^•^, and H_2_O_2_, as detailed in reactions (4), (5), (7), and (10). This is illustrated in Figure 1, which shows the time evolution of *G*(Fe^3+^) from our Monte Carlo simulations of Fricke dosimeter radiolysis by 300 MeV protons (LET~0.3 keV/µm) under aerated conditions at 25 °C, spanning ~1 ps to 200 s without dose–rate effects (see Section 4). As shown, the fastest Fe^3+^ formation occurs via Fe^2+^ oxidation by ^•^OH (reaction (4)), completed within ~10 µs, while the slowest process—the H_2_O_2_-driven Fenton-type reaction (7)—begins after ~0.1 s and completes by ~100 s.

As demonstrated in Equation (8), Fe^3+^ ion formation is most sensitive to factors that influence the “escape” free-radical yields, such as LET. As LET increases, the mean separation distance between “spurs” decreases, causing the isolated spur structure typical of low-LET irradiation to evolve into a continuous, dense cylindrical track (e.g., [9,21,22,38,39]). Under these conditions, more radicals are generated in close proximity, increasing the likelihood of their interaction to form molecular products or recombine to water. This enhanced intra-track radical–radical combination and recombination competes with the reactions between radicals and Fe^2+^, ultimately reducing *G*(Fe^3+^) as radicals are more likely to react with each other than with ferrous ions. Figure 1 illustrates these effects, showing the time evolution of *G*(Fe^3+^) from simulations of an aerated Fricke dosimeter irradiated with 1 MeV protons (LET~25 keV/µm) and 150 keV protons (LET~72 keV/µm), this latter corresponding to the Bragg peak region [40,41]. Recall that the Bragg peak refers to the increased concentration of ionizations as the incident particle slows down near the end of its track. The slower the particle’s speed, the longer it stays in proximity to a molecule, thereby increasing the probability of interactions and resulting in greater ionization and excitation [9,21,22]. As shown in the figure, *G*(Fe^3+^) decreases from ~15.35 to 9.85 to 8.15 molecules/100 eV as LET increases from ~0.3 to 25 to 72 keV/µm.

**Figure 2:**
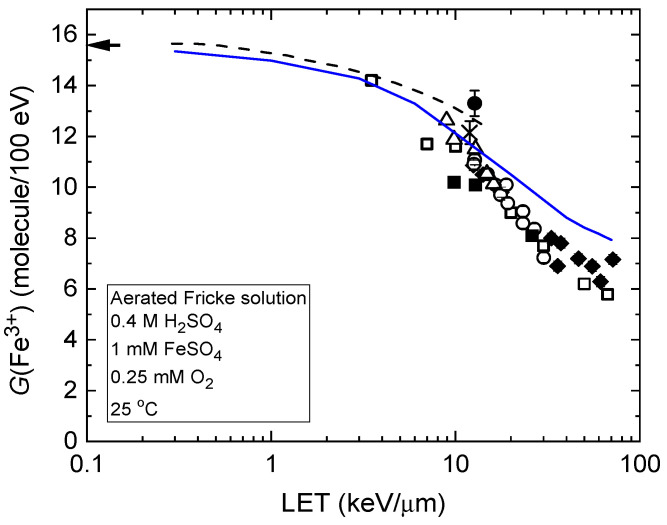
Ferric ion yield, *G*(Fe^3+^), from the radiolysis of air-saturated Fricke solutions as a function of LET, using protons with initial energies from 300 MeV (~0.3 keV/µm) to 150 keV (~72 keV/µm). The blue solid line shows *G*(Fe^3+^) values from our Monte Carlo simulations at ~100 s post-ionization (see Section 4). The dashed curve represents data from Autsavapromporn et al. [20] on the oxidation of ferrous sulfate by protons with LET values ranging from ~0.3 to 15 keV/µm. Experiment: ♦, Hart et al. [42]; o, Anderson and Hart [43]; ∆, Kochanny et al. [44]; !, Matsui et al. [45]; •, Sauer et al. [46]; □, LaVerne and Schuler [47]; ×, Elliot et al. [48]. The arrow in the top-left corner indicates the accepted *G*(Fe^3+^) value of 15.5 ± 0.2 molecules/100 eV for the aerated Fricke dosimeter irradiated with ^60^Co γ-rays or fast electrons. Adapted from Sepulveda et al. [37].

These LET effects are further illustrated in Figure 2, which presents the ferric ion yields as a function of LET for the radiolysis of the air-saturated Fricke dosimeter by protons with initial energies ranging from 300 MeV to 150 keV. As shown, the blue solid line representing *G*(Fe^3+^) from our Monte Carlo simulations (see Section 4) closely matches the broad experimental data on LET effects in Fricke dosimetry [42,43,44,45,46,47,48]. This agreement supports both the reliability of our simulation approach and the validity of the chemical reaction scheme used in this study to model the radiation chemistry of aerated Fricke solutions.

Furthermore, it is worthwhile to note that, at high LET, the production of Fe^3+^ is not solely determined by LET [21,49], but also depends on the charge of the irradiating particle. This is due to variations in the local energy density, which arise from differences in the microscopic track structure and the spatial distribution of the ejected secondary electrons [22]. Specifically, for the same LET, a particle with a higher charge exhibits a lower local energy density because of its higher velocity, which facilitates the escape of more radicals from the particle track to oxidize ferrous ions (e.g., see [50,51,52]). However, within the moderate LET range considered in this study, *G*(Fe^3+^) remains predominantly a singular function of LET, making it a valuable parameter for comparing *G*(Fe^3+^) across different radiation beam qualities.

### 2.4. Time Evolution of G(Fe^3+^) in the Radiolysis of the Fricke Dosimeter Within Gel-like Environments with Varying Viscosities

Fricke gel dosimetry involves incorporating the Fricke solution into a hydrogel matrix, which serves as a medium for radiation dose measurement. Gelatin and agarose are the most commonly used gelling agents, each providing distinct physical properties to the gel. Both gelatin-based and agarose-based Fricke gel dosimeters are considered tissue-equivalent, effectively simulating the radiation response of biological tissues [17,18]. This tissue-mimicking capability makes them particularly valuable for providing reliable dose measurements critical to optimizing radiation therapy treatment plans, as well as for advancing experimental research in radiation science. However, as discussed in Section 1, the dose distribution within Fricke gels is often compromised by the loss of spatial integrity, resulting from the diffusion of ferric ions within the gel matrix. This diffusion causes the radiation dose distribution to blur shortly after irradiation. In practice, this issue is typically mitigated by minimizing the time between irradiation and dosimeter analysis.

While this study does not seek to further quantify the impact of iron ion diffusion on post-irradiation three-dimensional dosimetric mapping, it instead focuses on examining the effects of gel viscosity on *G*(Fe^3+^) values from a radiation–chemical perspective. To this end, we systematically adjusted the diffusion coefficients of all radiolytic species produced during the radiolysis of the Fricke gel dosimeter—including both ferrous and ferric ions. This approach allows us to assess how changes in gel viscosity modulate the overall Fe^3+^ yield in the dosimeter.

**Figure 3:**
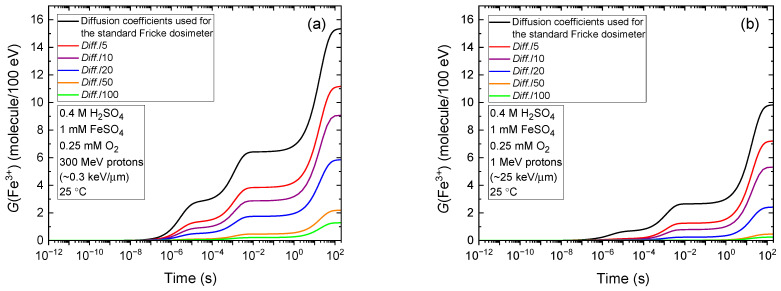
Temporal evolution of the ferric ion yield, *G*(Fe^3+^), calculated from Monte Carlo simulations of the radiolysis of aerated Fricke dosimeters in various gel-like environments irradiated by 300 MeV (LET~0.3 keV/µm) protons (**a**) and 1 MeV (LET~25 keV/µm) protons (**b**), over the time interval 1 ps to 200 s at 25 °C. The different curves represent increasing solution viscosity, modeled by reducing the diffusion coefficients of all radiolytic species—including ferrous and ferric ions (denoted as *Diff.* in the figure). Specifically, diffusion coefficients are decreased by factors of 5 (red), 10 (purple), 20 (blue), 50 (orange), and 100 (green), relative to those used for the standard Fricke dosimeter (black curve). Notably, the curves labeled *Diff.*/5 and *Diff.*/10 correspond to conditions representative of a gelatin-based Fricke gel dosimeter, in which the diffusion coefficient of ferric ions ranges from approximately 2.2 to 3.9 × 10^−10^ m^2^/s [14]. These values are roughly 5 to 10 times lower than the typical Fe^3+^ diffusion coefficient in standard Fricke solution, assumed to be ~2 × 10^−9^ m^2^/s in this study—slightly below the reported value of 6.04 × 10^−9^ m^2^/s in aqueous solution at infinite dilution [10,54]) at room temperature.

Figure 3a shows the evolution of *G*(Fe^3+^), as calculated from our Monte Carlo simulations of the radiolysis of air-saturated Fricke dosimeters irradiated with 300 MeV protons (LET~0.3 keV/µm), mimicking the low-LET conditions typical of Compton electrons generated by the absorption of ^60^Co γ rays in liquid water (see Section 4). In these simulations, the diffusion coefficients of all reactive species were systematically reduced by factors of 5, 10, 20, 50, and 100, relative to their standard values at 25 °C [10,53]. As shown, the Fricke yield decreases markedly—from 15.35 to 9.07 and 1.29 molecules/100 eV—for reductions by factors of 10 and 100, respectively. On the other hand, Figure 3b illustrates the effect of LET, showing the evolution of *G*(Fe^3+^) for the radiolysis induced by 1 MeV protons (LET~25 keV/µm). In this case, similar trends are observed with *G*(Fe^3+^) decreasing from 9.85 molecules/100 eV under standard conditions to 5.31 and 0.25 molecules/100 eV for diffusion coefficient reductions by factors of 10 and 100, respectively.

Quantitatively, the results in Figure 3a,b are significant and show that increasing the viscosity of Fricke gels to limit ferric ion diffusion and preserve spatial dose distribution stability also markedly reduces the ferric ion yield. This reduction, in turn, compromises both the accuracy and sensitivity of the dosimeter. The effect is particularly pronounced under high-LET irradiation, where the loss in yield and measurement precision is further exacerbated.

**Figure 4:**
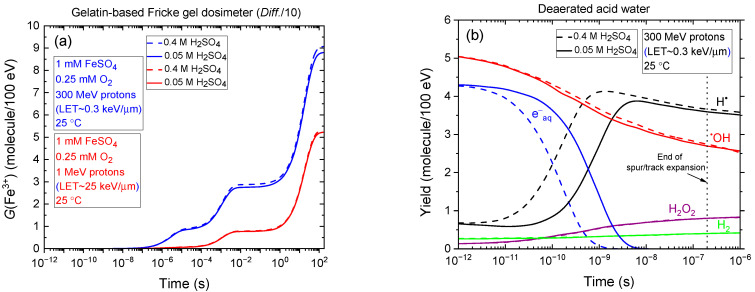
(**a**) Comparison of the temporal evolution of *G*(Fe^3+^) from Monte Carlo simulations of Fricke dosimeter radiolysis at two H_2_SO_4_ concentrations: 0.4 M and 0.05 M, at 25 °C. The simulations assume a gelatin-based Fricke dosimeter in which the diffusion coefficients for all reactive species—including ferrous and ferric ions—are reduced by a factor of 10 relative to those in the standard Fricke solution (*Diff.*/10). The comparison includes irradiation with 300 MeV (LET~0.3 keV/µm) and 1 MeV (LET~25 keV/µm) protons. (**b**) Time evolution of the yields (in molecules per 100 eV) of radiolytically produced species—e^−^_aq_ (blue), H^•^ (black), H_2_ (green), ^•^OH (red), and H_2_O_2_ (purple), obtained from Monte Carlo simulations of the radiolysis of acid, air-free water irradiated by 300 MeV protons at 25 °C, over the time interval ~1 ps to 1 µs (see Section 4). Solid lines correspond to 0.05 M H_2_SO_4_, while dashed lines represent 0.4 M H_2_SO_4_. The vertical dotted line at ~0.2 µs marks the end of spur/track expansion, denoting the transition from nonhomogeneous track kinetics to homogeneous bulk-phase chemistry [27]. As shown, all e^−^_aq_ species are removed in spur/track reactions—primarily through conversion to H^•^ atoms via reaction (2) with hydronium ions (H_3_O^+^)—well before the dissipation of the radiation track structure.

As previously noted, 0.4 M sulfuric acid can degrade the Fricke gel matrix. To preserve the spatial integrity of the dose distribution, lower acid concentrations—such as 0.05 M—are commonly employed in gel-based dosimeters. It is therefore of interest to compare the temporal evolution of *G*(Fe^3+^) obtained from our Monte Carlo simulations at these two sulfuric acid concentrations: 0.4 and 0.05 M. This comparison, shown in Figure 4a, includes irradiation with 300 MeV (LET~0.3 keV/µm) and 1 MeV (LET~25 keV/µm) protons, assuming a gelatin-based Fricke gel dosimeter in which the diffusion coefficients of reactive species are reduced by a factor of ten relative to the standard Fricke solution. As shown, the *G*(Fe^3+^) versus time curves are nearly superimposable—particularly under high-LET conditions—indicating that the Fricke yields at ~100 s are essentially identical for both acid concentrations. According to Equation (8), this suggests that the primary (“escape”) yields of radicals and molecular products from the radiolysis of acidic water remain largely unaffected by the change in sulfuric acid concentration. This conclusion is further supported by Figure 4b, which compares the time evolution of various radiolytic species yields in deaerated water irradiated by 300 MeV protons at 25 °C. As observed, the “escape” yields at ~0.2 µs—corresponding to the end of spur/track expansion [27]—are virtually identical for both 0.4 and 0.05 M H_2_SO_4_.

**Figure 5:**
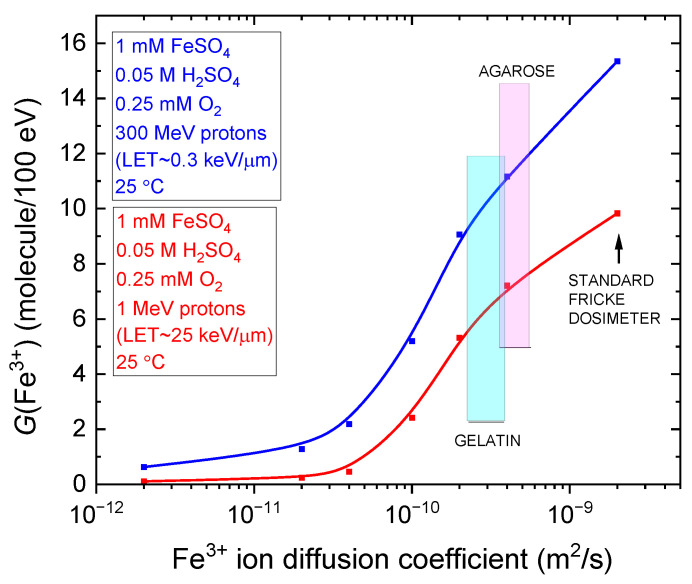
Plot of Fricke yields, *G*(Fe^3+^), as a function of the ferric ion diffusion coefficient in the radiolysis of the aerated Fricke dosimeter embedded in gel-like environments of varying viscosity, including the standard Fricke dosimeter. The *G*(Fe^3+^) values were extracted at ~100 s from the simulations presented in Figure 3a,b for both 300 MeV (LET~0.3 keV/µm) and 1 MeV (LET~25 keV/µm) proton irradiations at 25 °C for the standard Fricke dosimeter, as well as for the curves labeled *Diff.*/5, *Diff.*/10, *Diff.*/20, *Diff.*/50, and *Diff.*/100. The value for *Diff.*/1000 is also shown, although it was omitted from Figure 3a,b for clarity. Vertical shaded bands highlight the literature-reported ranges of Fe^3+^ diffusion coefficients for gelatin-based (cyan; 2.2–3.9 × 10^−10^ m^2^/s) and agarose-based (magenta; 3.6–5.6 × 10^−10^ m^2^/s) Fricke gel dosimeters, as summarized in Table 2 of [14], for visual emphasis. Note that the viscosity of these systems depends on the concentration of the gelling agent.

Finally, Figure 5 shows the variation in *G*(Fe^3+^) as a function of the ferric ion diffusion coefficient in the radiolysis of the aerated Fricke dosimeter embedded in gel-like environments with varying viscosities. Monte Carlo simulations were conducted at a sulfuric acid concentration of 0.05 M, using 300 MeV (LET~0.3 keV/µm) and 1 MeV (LET~25 keV/µm) protons at 25 °C. The diffusion coefficients span from the typical value for Fe^3+^ in the standard Fricke solution (~2 × 10^−9^ m^2^/s) down to a value reduced by a factor of 1000, reflecting extreme viscosity conditions. As shown, *G*(Fe^3+^) remains relatively unchanged as the diffusion coefficient increases, until it reaches ~4 × 10^−11^ m^2^/s for both low- and high-LET proton irradiations. Beyond this threshold, *G*(Fe^3+^) rises sharply, reaching 15.35 and 9.85 molecules/100 eV for the standard Fricke dosimeter under 300 MeV and 1 MeV proton irradiation, respectively, as previously noted. In the same figure, the ferric ion diffusion coefficients for gelatin-based and agarose-based Fricke gel dosimeters are indicated at 2.2–3.9 × 10^−10^ m^2^/s and 3.6–5.6 × 10^−10^ m^2^/s [14], respectively. As observed, ferric ion diffusion in agarose gels is slightly higher—by roughly a factor of two—compared with gelatin gels [18,55].

Figure 5 is particularly insightful, as it quantitatively demonstrates that an optimal Fricke gel dosimeter must balance two critical factors: maximizing the Fe^3+^ yield to ensure high dosimetric sensitivity and accuracy, while minimizing the Fe^3+^ diffusion coefficient to preserve the spatial integrity of 3D dose distributions post-irradiation. Striking this balance is essential for achieving both precise measurement and stable, well-defined radiation dose profiles—key requirements for reliable analysis and interpretation of irradiation effects.

## 3. Conclusions

In this study, we investigated, from a radiation–chemical perspective, the variation in ferric ion yield in both the standard aqueous Fricke dosimeter and gel-like Fricke systems with varying viscosities. The analysis was conducted under two distinct LET conditions—~0.3 keV/µm and 25 keV/µm—using 300 MeV and 1 MeV protons, respectively, as radiation sources.

Using the Monte Carlo track chemistry code IONLYS-IRT, we evaluated the impact of gel viscosity on the Fricke yield, *G*(Fe^3+^), by systematically modifying the diffusion coefficients of all radiolytic species generated during the radiolysis of the Fricke gel dosimeter, including Fe^2+^ and Fe^3+^ ions. Increasing the gel viscosity reduces ferric ion diffusion and helps preserve the spatial integrity of 3D dose distributions post-irradiation; however, it also leads to a marked decrease in *G*(Fe^3+^). This reduction compromises the dosimeter’s sensitivity and accuracy, particularly under high-LET irradiation. Our findings provide valuable quantitative evidence that an optimal Fricke gel dosimeter must strike a balance between two competing requirements: maximizing *G*(Fe^3+^) and minimizing Fe^3+^ ion diffusion. Achieving this balance is essential for ensuring precise dosimetric measurements and stable, spatially resolved dose profiles critical for an accurate analysis of irradiation effects.

Additionally, simulations were performed using a reduced sulfuric acid concentration of 0.05 M, in place of the standard 0.4 M typically used in aqueous Fricke dosimeters. This lower concentration—commonly employed in gel-based systems to prevent degradation of the gel matrix—had minimal impact on the Fricke yield, which remained nearly unchanged under both low- and high-LET irradiation.

Complementing the predominantly empirical approaches found in the literature, which focus on the fundamental principles of Fricke gel dosimetry and its underlying chemistry, this study demonstrates the value of Monte Carlo simulations as a powerful quantitative tool for modeling viscosity effects in Fricke gel radiolysis.

These simulations are effective across both low- and high-LET conditions and provide critical insights into the physicochemical interactions within the gel matrix. Such insights support the optimization of dosimeter formulations by enhancing stability, preserving spatial resolution, and maximizing sensitivity and accuracy, while minimizing the post-irradiation diffusion of ferric ions.

Ongoing Monte Carlo simulations in our laboratory aim to investigate the impact of dose–rate effects on Fricke yields in gel dosimeters of varying viscosities, under both low- and high-LET irradiation conditions. These studies are particularly relevant to the ultra-high dose rates characteristic of the “FLASH effect” observed in radiobiology and radiotherapy (e.g., see [56,57,58,59,60,61] and references therein). FLASH radiotherapy is now well recognized for leveraging a remarkable biological phenomenon in which radiation doses are delivered at rates several orders of magnitude higher than those used in conventional therapy—typically exceeding 40–100 Gy/s. This approach has been shown to substantially reduce normal tissue toxicity while maintaining, or even enhancing, tumor control, thereby offering a promising therapeutic advantage. Although the underlying mechanisms remain under active investigation, radical chemistry and oxygen dynamics are believed to play key roles—areas in which Fricke-based dosimetry and track-structure simulations may offer valuable insight.

## 4. Materials and Methods

This study was carried out using an extended version of our Monte Carlo track chemistry simulation code, IONLYS-IRT [22,53,62], to model the radiolysis of aerated Fricke solutions of varying viscosity at 25 °C, under irradiation by both low- and high-LET protons. This approach enabled a more detailed investigation of how viscosity influences Fricke gel dosimeters, viewed from a radiation–chemical perspective. Although the full details of this version of the code have been published previously (e.g., see [10,20,37,63]), a brief summary of its key features is provided below.

Our event-by-event IONLYS program models the early physical and physicochemical stages of radiation action in a 3D aqueous environment, simulating the spatiotemporal evolution of proton tracks up to ~1 ps. The program consists of two main modules: TRACPRO, which handles proton transport, and TRACELE, which simulates the transport of secondary electrons (δ-rays). It generates the complex, nonhomogeneous spatial distribution of radiolytic species at the end of the physicochemical stage—including e^−^_aq_, H_3_O^+^, OH^−^, H^•^, H_2_, ^•^OH, H_2_O_2_, HO_2_^•^/O_2_^•−^, O(^1^*D*), ^•^O^•^(^3^*P*), O^•−^, among others [22])—which serves as the starting point for the subsequent chemical stage.

In the third stage (>1 ps), radiolytic species diffuse according to their diffusion coefficients and undergo reactions either with one another or with dissolved solutes present at the time of irradiation (e.g., O_2_ in aerated Fricke solution and ferrous ions). This stage is modeled using our IRT program [53], which implements the “independent reaction times” (IRT) method [64,65,66]—a stochastic approach that computes reaction times without explicitly tracking the trajectories of diffusing species. The accuracy of this method has been validated through comparisons with full random-flight Monte Carlo simulations that follow particle trajectories [67]. Additionally, the IRT program is well-suited for modeling long-term chemistry, when tracks have dissipated and the radiolytic products are homogeneously distributed in the solution, as in Fricke dosimeter simulations, where ferric ions continue to form over time scales extending up to ~100 s (see Figure 3).

The chemical reaction scheme, rate constants, and diffusion coefficients used in our IONLYS-IRT code for simulating the radiolysis of Fricke solutions are consistent with those reported in previous studies [10,37,63]. In brief, the reaction scheme for pure water radiolysis (see [22] for a review) was extended to include reactions involving HSO_4_^−^, SO_4_^2−^, SO_4_^•−^, and S_2_O_8_^2−^ in irradiated sulfuric acid solutions, as detailed in Table 1 of [20]. To model the chemistry of the Fricke dosimeter, the IRT module incorporates reactions (4), (5), (7), and (10), which describe the oxidation of Fe^2+^ by various radiolytically generated oxidants. Under the irradiation conditions considered in this study, the concentrations of radiolytic products remain low relative to the background levels of H^+^ (~0.4 M), Fe^2+^ (1 mM), and O_2_ (~0.25 mM), allowing their reactions to be treated as pseudo-first-order processes within the IRT framework.

Our IRT program also accounts for the effect of ionic strength on all ionic reactions, with the exception of the bimolecular self-recombination of e^−^_aq_, which is known to be unaffected by ionic strength [68]. Rate constant corrections were applied as described in [37,69]. Additionally, the “direct” action of ionizing radiation on solutes was neglected—a reasonable approximation given their relatively low concentrations under the conditions considered in this study.

Diffusion, driven by the random thermal motion of particles, is quantified by the diffusion coefficient. According to the Stokes–Einstein relation, which describes the diffusion of spherical particles in a homogeneous fluid, the diffusion coefficient is inversely proportional to the fluid’s viscosity. Thus, increasing viscosity results in a corresponding decrease in diffusion. This fundamental relationship is central to our study. Beginning with the standard aqueous Fricke dosimeter, we extended this concept to Fricke gel dosimeters with varying viscosities by systematically adjusting the diffusion coefficients of all radiolytic species generated during irradiation, including both ferrous and ferric ions. This approach enables us to investigate the influence of viscosity on the radiation chemistry of Fricke-infused gel dosimeters, using gelatin and agarose as representative gelling agents [17,18,19].

This study focuses solely on results obtained at 25 °C. All simulations were performed using short proton track segments (typically ~10–150 µm) of 300 MeV and 1 MeV protons, corresponding to LET values of ~0.3 and 25 keV/µm, respectively. These LET values are consistent with data reported by Watt [40] and the recommendations of ICRU 49 [41] for liquid water (density = 1 g/cm^3^). We assumed that the densities of the various ferrous sulfate gel dosimeters considered were similar to that of water, typically ranging from ~1.0 to 1.3 g/cm^3^. Over the simulated track segments, proton energy and LET were well defined and remained nearly constant. For our irradiation conditions, the number of simulated proton “histories” (usually between 5 and 100, depending on LET) was selected to minimize statistical variations in the average chemical yields while keeping reasonable computation times. In all simulations, the temporal evolution of *G*(Fe^3+^) was followed up to ~200 s.

## Data Availability

Data generated or analyzed during this study are provided in full within the article. For further inquiries, please contact the authors directly.
